# Microbiological Screening of 5-Functionalized Pyrazoles for the Future Development of Optimized Pyrazole-Based Delivery Systems

**DOI:** 10.3390/pharmaceutics14091770

**Published:** 2022-08-24

**Authors:** Chiara Brullo, Debora Caviglia, Andrea Spallarossa, Silvana Alfei, Scott G. Franzblau, Bruno Tasso, Anna Maria Schito

**Affiliations:** 1 Department of Pharmacy (DIFAR), Section of Medicinal Chemistry, University of Genoa, Viale Benedetto XV 3, 16132 Genoa, Italy; 2 Department of Surgical Sciences and Integrated Diagnostics (DISC), University of Genoa, Viale Benedetto XV 6, 16132 Genoa, Italy; 3 Department of Pharmacy (DIFAR), Section of Organic Chemistry, University of Genoa, Viale Cembrano 4, 16148 Genoa, Italy; 4 Institute for Tuberculosis Research, College of Pharmacy, University of Illinois at Chicago, 833 South Wood Street, Chicago, IL 60612, USA

**Keywords:** pyrazole, 5-aminopyrazoles, antibacterial activity, Gram-positive species, *Staphylococcus* genus, in silico pharmacokinetic properties prediction

## Abstract

The pyrazole ring represents a widely applied chemical scaffold in medicinal chemistry research and we have observed that the physicochemical and biological features of highly substituted pyrazoles can be successfully improved by their encapsulation in dendrimer nanoparticles (NPs). For the future development of new optimized antibacterial delivery systems, we report the synthesis and biological evaluation of 5-amino functionalized pyrazole library (compounds **2**–**7**). In detail, new derivatives **2**–**7** were differently decorated in C3, C4 and C5 positions. An in silico study predicted pyrazoles **2**–**7** to exert good drug-like and pharmacokinetic properties. Compounds **3c** and **4b** were endowed with moderate, but nanotechnologically improvable activity against multidrug-resistant (MDR) clinical isolates of Gram-positive species, especially of the *Staphylococcus* genus (MICs = 32–64 µg/mL). In addition, derivatives **3c** and **4a** showed moderate activities against *Mycobacterium tuberculosis* and **4a** evidenced activity also against MDR strains. Overall, the collected evidence supported that, upon nano-formulation with proper polymer matrices, the new synthesized compounds could provide new pyrazole-based drug delivery systems with an enhanced and enlarged-spectrum of antibacterial activity.

## 1. Introduction

**Figure 1:**
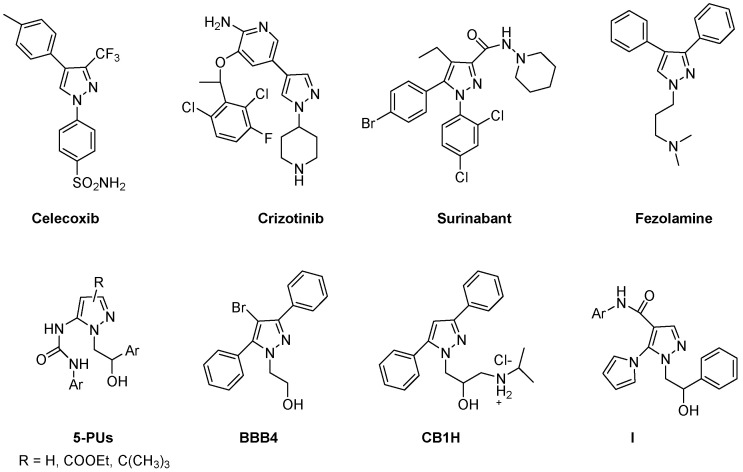
Chemical structure of some drugs endowed with the pyrazole structure (Celecoxib, Crizotinib, Surinabant, Fezolamine) and **5-PUs**, **BBB4**, **CB1H** and **I** previously reported as anticancer/anti-inflammatory, antibacterial and anti-*Mycobacterium* compounds, respectively.

The five-membered heterocycle pyrazole ring represents a widely applied chemical scaffold in medicinal chemistry research. Several pyrazole derivatives show a broad spectrum of biological properties, such as antimicrobial [1], analgesic [2], antifungal [3], anti-inflammatory [4], and antioxidant activities [5]. Collectively, the pyrazole nucleus possesses almost all types of pharmacological activities, and throughout the years, many researchers have studied its skeleton both chemically and biologically. Currently, the pyrazole nucleus is present in numerous pharmacological agents belonging to different therapeutic categories (Figure 1), as anti-inflammatory/analgesic compounds (Celecoxib, Tepoxalin, Betazole), anticancer (Crizotimib), antiobesity (Surinabant, Difenamizole), antidepressant and tranquillizer (Fezolamine, Mepiprazole) and many others, confirming the pharmacological potential of this heterocyclic ring [6]. Particularly, 5-pyrazolyl-ureas (5-PUs) (Figure 1) are reported and studied for the treatment of different microbial infections caused by bacteria or protozoa of the genera *Plasmodium, Toxoplasma*, and others [7] or to treat cancer (e.g., antiangiogenic agents and Src, p38-MAPK and TrKa inhibitors) [7,8,9,10]. Notably, 5-PUs bearing a 2-hydroxy-2-phenylethyl chain at the N1 position were identified as effective anticancer, anti-inflammatory, and anti-*Mycobacterium* agents [8,9,10,11,12,13].

Additionally, we have observed that the water solubility and antibacterial effects of two differently functionalized pyrazoles synthetized by us (**BBB4** and **CB1H**) (Figure 1) were remarkably improved by their encapsulation in polyester-based dendrimer nanoparticles (NPs) and in styrene-based copolymer NPs [6,14]. In particular, the obtained BBB4-G4K NPs and CB1H-P7 NPs were able to contrast multidrug-resistant (MDR) pathogenic species, also including clinical isolates particularly difficult to treat, due to the onset of resistance versus the most potent antibiotics, such as carbapenems and colistin [15,16]. Additionally, the developed encapsulated formulations showed lower cytotoxicity in human cells and higher selectivity indices than the free pyrazole compounds [15,16]. On the other hand, other 5-pyrrolyl-pyrazoles **I** (Figure 1), characterized as our previous 5-PUs by an N1-hydroxy-2-phenylethyl chain, evidenced some anti-*Mycobacterium* activity [13]. 

**Figure 2:**
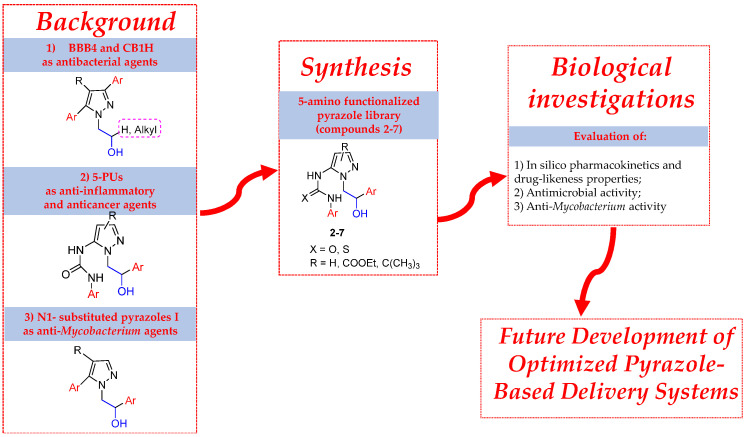
Workflow of the applied strategies leading to the design, biological evaluation and future development of novel pyrazole compounds **2**–**7**.

In the current scenario of bacterial infections, those caused by bacteria of the ESKAPE (Enterococcus faecium, Staphylococcus aureus, Klebsiella pneumonia, Acinetobacter baumannii, Pseudomonas aeruginosa, Enterobacter) family, which are recognized as dangerous superbugs by the Infectious Diseases Society of America (IDSA), are becoming a global concern because they are almost untreatable [6]. Unfortunately, ESKAPE bacteria are endowed with an increasing tendency to develop MDR to the bactericidal effect of conventional antibiotics, which are no longer effective and need substitution with more efficient new antibacterial agents suitable for clinical application [6]. To meet the global urgency of new antimicrobial compounds, the aim of this work is to investigate the antibacterial/anti-Mycobacterium activity of a small library of 5-amino functionalized pyrazoles (compounds **2**–**7,** see the workflow of the whole study in Figure 2), structurally related to previously reported **BBB4**, **CB1H, 5-PUs** and **I**. To reduce the lipophilicity (and therefore increase water solubility) and preserve antimicrobial activity, the new pyrazole derivatives do not bear the 3,5-diphenyl groups but share with the parent compounds the N1 2-hydroxy-2-phenylethyl chain identified in previous studies as a relevant pharmacophoric portion [11,12,13]. Particularly, compounds **2** and **3** (Table 1) are characterized by an ureido moiety in the 5 position and a carboxyethyl (derivatives **2a**–**d**) or a tert-butyl (derivatives **3a**–**d**) substituent at C4 and C3, respectively. Compounds **4**–**6** (Table 1) share with derivatives **2** the 4-carboxyethyl substituent but bear at C5 a benzoyl-thiourea (derivatives **4a**–**d**), an amide (derivatives **5a**–**d**) or a sulfonamide function (derivative **6**). Finally, compound **7** is a positional isomer of derivative **4c** characterized by a carboxyethyl substituent moved from position 4 to position 3. Within each series of compounds, the substituent at position 5 was varied to define structure–activity relationships (SAR) on the effect of this functionalization on antibacterial/anti-Mycobacterium activity. In fact, except for compounds **5a**, all compounds bear at different positions (ortho, meta, para) of the phenyl ring in the functional group at position 5 a fluorine atom or a trifluoromethyl group to increase lipophilicity.

## 2. Results and Discussion

### 2.1. Chemistry 

**Scheme 1:**
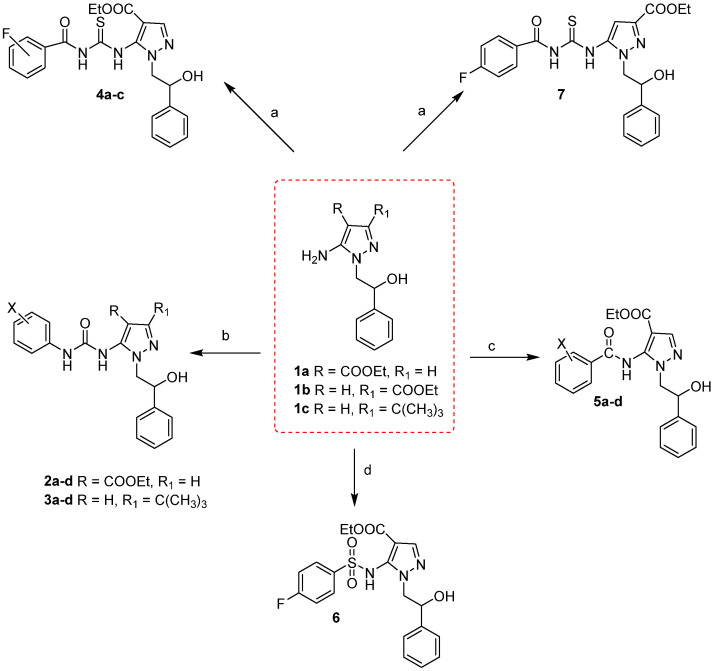
Synthesis of compounds **2–7**. Reagents and conditions: (**a**) suitable benzoyl isothiocyanates [18], anh. THF, reflux, 12 h; (**b**) suitable phenyl isocyanates, anh. toluene, reflux, 6 h; (**c**) suitable benzoyl chlorides, TEA, anh. THF, reflux, 18 h; (**d**) 4-fluorobenzenesulfonyl chloride, TEA, anh. THF, reflux, 3 days.

Compounds **2**–**7** were synthesized by a divergent synthetic protocol starting from the pyrazole key intermediates **1a**–**c** (Scheme 1) [12,17] by functionalization of the 5-amino group with different electrophilic reagents as isocyanates, benzoylisocyanates, benzoyl chlorides and 4-fluorobenzenesulfonyl chloride. In general, reaction yields are good for compounds **2**–**4** (from 42% to 51%), and lower for compounds **5** and **6** (from 23% to 61%). In general, compounds derived from pyrazole **1a** (substituted in C4 position) were obtained with yields higher than compounds obtained from pyrazoles **1b** and **1c** (substituted at the C3 position), as demonstrated comparing the reaction yields of compound **4c** with its isomer **7** (51% versus 37%), evidencing a different reactivity versus electrophilic reagents of amino group at the 5 position of pyrazole **1a** withrespect to pyrazoles **1b**,**c**. As previously reported [11,12], compounds **2a**–**d** and **3a**–**d**, characterized by a urea moiety at the 5 position of the pyrazole nucleus, were synthesized by refluxing in anhydrous toluene **1a** or **1c** with a small excess (1.1. equivalents) of suitable phenyl isocyanate for 6 h. Compounds **4a**–**c** and **7**, in which the urea moiety was transformed into thiourea, were obtained by reacting pyrazoles **1a,b** with the suitable benzoyl isothiocyanate, previously prepared modifying a method from the literature [18], in anhydrous THF at reflux for 12 h. Finally, amides **5a**-**e** and the sulphonamide **6** were obtained by condensing **1** with benzoyl chlorides (for derivatives **5**) or 4-fluorobenzenesulfonyl chloride (for derivative **6**) in anhydrous THF in the presence of triethylamine (TEA) at reflux for 18 h (compounds **5**) or 3 days (compound **6**).

### 2.2. Pharmacokinetic Properties and Druglikeness Prediction

To evaluate the pharmaceutical relevance of this 5-amino functionalized library, the pharmacokinetics properties, as well as the druglikeness of compounds **2**–**7** were calculated by SwissADME [19]. Collectively, an in silico study predicted these compounds to exert good drug-like and pharmacokinetic properties, particularly regarding physicochemical properties, lipophilicity and water solubility. Interestingly, no violations of the Lipinski rules were detected, as well as any pan assay interference compound (PAINS) alerts were reported. Exclusively for compounds **4** and **7,** the presence of the thiocarbonyl functionality at the C5 position was spotted as problematic fragment(s) according to the Brenk filters [20]. Overall, the collected data support the pharmaceutical potentials of derivatives **3** and **4**.

In detail, as reported in Table 2 and Table 3, the calculated physicochemical parameters (i.e., LogP range: 2.74–4.5; MW range: 396.46–462.42 g/mol; TPSA range: 79–137 Å^2^; Fraction Csp3 range: 0.18–0.3; number of rotatable bonds: 8–11) indicated a good oral bioavailability. In addition, compounds **2a**–**c** and **5a** were predicted as water-soluble, whereas the other derivatives moderately water-soluble. Furthermore, the predicted gastrointestinal (GI) absorption would be high for derivatives **2,3,5,6** whereas thioureido compounds **4** and **7** would be poorly adsorbed in the GI tract. None of the analyzed compounds were predicted to penetrate the blood–brain barrier (BBB). According to the calculations, tested derivatives can inhibit some of the cytochrome (CYP) isoforms (1A2, 2C19, 2C9, 2D6, 3A4), but only **2a**–**c** and **3c** are substrates of Pgp. 

### 2.3. Antimicrobial Activity

Supported by the promising pharmacokinetic and druglikeness properties predicted for derivatives **2**–**7** and based on the structural similarities with the previous compounds **BBB4**, **CB1H** and **I**, the antibacterial activity of all compounds was preliminarily evaluated. Out of this initial screening, which established the inactivity of all compounds against bacteria of the Gram-negative species, compounds **3a**–**c** and **4a**–**c**, characterized by a urea moiety (**3**) or thiourea function (**4**) at the C5 position, evidenced in some cases promising antibacterial profiles. Based on these results, they have been selected for further evaluation against twenty-one clinical and MDR isolates of the genera *Staphylococcus* and *Enterococcus* (Table 4). 

Compounds were considered inactive against a specific strain when MIC values higher than 128 µg/mL were observed. Thus, compound **3c** showed widespread activity (MICs = 32–64 µg/mL) on selected isolates, emerging ineffective only against *E. faecalis* 365, and less clinically relevant enterococci and staphylococci, including *E. gallinarum* 150, *E. casseliflavus* 184, *S. simulans* 163 and *S. haemolyticus* 193 strains. Interestingly, the substitution pattern of the urea phenyl ring deeply affects the antibacterial properties of the compounds. Specifically, 5-PUs **3a** (*ortho*-F substituted) and **3b** (*meta*-F substituted) showed reduced activity in comparison with their 4-fluorophenyl analogue **3c**, still capable of affecting the duplication of strains not influenced by **3c** (namely, *E. gallinarum* 150 and *E. casseliflavus* 184). Collectively, compound **3c** demonstrated significant antibacterial activity against practically all the isolates considered, regardless of their resistance patterns. Particularly, as reported in Table 4, compound **3c** displayed MICs = 32–64 µg/mL against all staphylococci considered in this study, except for one isolate, and MICs = 64 µg/mL against all *E. faecium* isolates tested, two out of three isolates of *E. faecalis* and one out of three isolates of enterococci. Derivatives **4** emerged to be inactive against all strains of the *Enterococcus* genus, being moderately active (MICs = 32–64 µg/mL) on some isolates of the *Staphylococcus* genus (namely, *S. aureus* 18 and 187, *S. epidermidis* 180 and 181 and *S. lugdunensis* 129 (**4b**)*, S. aureus* 187 and *S. lugdunensis* 129 (**4c**) and only *S. lugdunensis* 129 (**4a**)). Overall, compound **3c** and, although with a very narrow spectrum on staphylococci, compound **4b** resulted as the most promising molecules between compounds **3a**–**c** and **4a**–**c** for future nano-formulation studies. To further define their potency, the antibacterial effects of **3c** and **4b** were compared with those of commonly used antibiotics (Table 5). Both compounds showed similar or improved activities in comparison to the reference antibiotics (i.e., ampicillin, ciprofloxacin and oxacillin) against sensitive isolates, thus supporting the pharmaceutical potentials of these pyrazole derivatives as novel antibacterial agents. 

Compounds **3c** (*para*-F substituted) and **4b** (*meta*-F substituted), characterized by a urea or thiourea function at C5 of the pyrazole nucleus, respectively, were found as the most active derivatives. On the contrary, the presence in this position of an amide (compounds **5**) or sulfonamide (compounds **6**) moiety negatively affected the antibacterial properties. Furthermore, relevant structural features for antibacterial activity include: (i) the carboxyethyl group at C4 and not at C3 (compare **4a** with **7**, Table 4); (ii) a *tert*-butyl substituent at C3 (as in compounds **3**). However, the simultaneous presence of the carboxyethyl substituent at C4 and of a urea function at C5 (as in compounds **2**) caused a reduction in biological activity. Finally, compounds in which the aromatic ring at the 5 position is not substituted (as in **5a**) or presented a trifluoromethyl group (as in **3d** and **5d**) showed decreased activity with respect to fluoro-substituted analogues.

Data reported in Table 5 established that, both compounds **3c** and **4b** show promise in the development of new drug delivery systems to specifically counteract infections sustained by staphylococci, where both ciprofloxacin and most beta-lactam drugs are no longer active. Additionally, compound **3c** also proved to be promising against enterococci isolates that are difficult to inhibit with ampicillin.

### 2.4. Anti-Mycobacterium Activity

All compounds were preliminary screened at 50 μg/mL for growth inhibition of replicating cultures of *Mycobacterium tuberculosis* using the Microplate Alamar Blue Assay (MABA). This concentration was selected according to literature data [13] and is normally used in screening protocols to test compounds against *Mycobacterium tubercolosis*. Additionally, pyrazoles **2**–**7** were tested against low oxygen-adapted *M. tuberculosis* containing a luxABCDE reporter under hypoxia using the low oxygen recovery assay (LORA) to assess compound activity against non-replicating cultures [22]. Most compounds **2**–**7** proved to be inactive, showing a percentage of inhibition concentration lower than 50% in the MABA test. Conversely, selected derivatives **3** and **4** evidenced a moderate activity (Table 6). Particularly, compounds **3c** and **4a**, characterized by a fluorophenyl substituent in *para* (**3c**) or *ortho* (**4a**) position, evidenced the most promising results, being able to block the duplication of *M. tuberculosis* at 50 μg/mL concentration. The derived MIC values for **3c** and **4a** were 35.8 and 39.3 μg/mL, respectively (Table 6). Unfortunately, none of the tested derivatives showed significant inhibition in the LORA test.

Finally, compounds 2-7 underwent extensive preliminary screening to assess their potential to inhibit the growth of MDR *M. tuberculosis* strains, responsible for a form of tuberculosis resistant to at least two first-line drugs for tuberculosis (Lilly TB Drug Discovery Initiative) [23]. From this large screening, only compound 4a evidenced weak activity at 20 μM concentration (31% of inhibition), representing a good starting point to develop new agents able to counteract MDR *M. tuberculosis*.

## 3. Materials and Methods

### 3.1. General Information

All chemicals were purchased by Chiminord srl and Merk (Aldrich Chemical) (Milan, Italy). Solvents were reagent grade. All commercial reagents were used without further purification. Aluminium-backed silica gel plates (Merck DC-Alufolien Kie-selgel 60 F254, Darmstad, Germany), were used in thin-layer chromatography (TLC) for routine monitoring of the reaction course. Merck silica gel, 230–400 mesh, was used for chromatography. Flash chromatography was performed using Isolera one instrument (Biotage, Uppsala, Sweden) using Silicagel column. Melting points are not “corrected” and were obtained with a Buchi M-560 instrument (Buchi instruments, Flawil, Switzerland). IR spectra were recorded with a Perkin-Elmer 398 spectrophotometer (Perkin-Elmer, Milan, Italy) for compounds **4**, **5a**–**c** and **6** or Spectrum Two FT-IR Spectrometer (PerkinElmer, Inc., Waltham, MA, USA) for compounds **5d** and **7**. The 1H NMR spectra were recorded on a Varian Gemini 200 (200 MHz, Varian Gemini, Palo Alto, CA, USA); chemical shifts are reported as δ (ppm) relative to tetramethylsilane (TMS) as internal standard; signals were characterized as s (singlet), d (doublet), t (triplet), n t (near triplet), q (quartet), m (multiplet), br s (broad signal); J were reported in Hz (see Supporting Material). The 13C NMR spectra were recorded on a JEOL JNM ECZ-400S/L1 (101 MHz, Tokyo, Japan). 

Elemental analysis was determined with an elemental analyzer EA 1110 (Fison-Instruments, Milan, Italy); products were considered pure when the difference between the calculated and found values is ± than 0.4 (see Table 7).

### 3.2. Synthesis of Compounds ***2a-d*** and ***3a-d***

A mixture of 5-amino-pyrazoles 1a [17] or 1c [12] (10 mmol) and the suitable phenyl isocyanates (11 mmol) in anhydrous toluene (70 mL) was heated at reflux for 12 h. After cooling to room temperature, the organic solution was washed with 3 M HCl (2 × 20 mL), water (10 mL), dried (MgSO4) and evaporated under reduced pressure. The obtained crude oil was crystallized by adding a solution of diethyl ether/petroleum ether (b.p. 50–60 °C) (1:1) or purified by flash chromatography using a mixture of diethyl ether/petroleum ether (b.p. 50–60 °C) 1/1 as the eluent. Analytical data of compounds **2a**–**d** and **3a**–**d** are previously reported [11,12].

#### 3.2.1. Synthesis of Compounds **4a**–**c**

A mixture of 5-amino-pyrazole **1a** (275 mg, 1 mmol) [17] and the proper benzoyl isothiocyanate (1 mmol), previously prepared modifying a method from the literature [18], in anhydrous THF (10 mL) was refluxed for 12 h. After cooling to room temperature, the solution was concentrated under reduced pressure. The crude crystallized by adding a solution of diethyl ether/petroleum ether (b.p. 50–60 °C) (1:1) or purified by flash chromatography using a mixture of diethyl ether/petroleum ether (b.p. 50–60 °C) 1/1 as the eluent.

##### Synthesis of 2-Fluorobenzoyl isothiocyanate, 3-Fluorobenzoyl isothiocyanate and 4-Fluorobenzoyl isothiocyanate

To a suspension of anhydrous potassium thiocyanate (1.62 g, 16.7 mmoles) in an. toluene (15 mL), a solution of the suitable fluorobenzoyl chloride (13.9 mmoles) in an. toluene (5 mL) was added dropwise. The reaction was heated to 70 °C for 7 h. After the reaction was complete, the mixture was cooled to room temperature and filtered through a Celite pad. The solvent was evaporated to give an oil that crystallized upon standing in a refrigerator. The product was used without further purification (yield: 79%, 81% and 82% for 2-Fluorobenzoyl isothiocyanate, 3-Fluorobenzoyl isothiocyanate and 4-Fluorobenzoyl isothiocyanate, respectively).

*Ethyl 5-(3-(2-fluorobenzoyl)-thioureido)-1-(2-hydroxy-2-phenylethyl)-1H-pyrazole-4-carboxylate***4a**. White solid. Yield 42%; mp 150–151 °C. ^1^H-NMR (400 MHz, CDCl_3_): δ 1.20 (t, *J* = 7.0, 3H, CH_3_), 3.92–4.10 (m, 4H, CH_2_O + CH_2_N), 4.87–4.90 (m, 1H, *CH*OH), 5.92 (br s, 1H, exchangeable), 7.19–7.43 (m, 9H Ar), 7.86 (s, 1H, H-3), 8.05 (br s, 1H, exchangeable), 12.00 (br s, 1H, exchangeable). ^13^C NMR (101 MHz, CDCl_3_): δ 177.0, 164.6, 161.4, 159.2, 142.3, 140.2, 132.2, 130.0, 128.4, 127.2, 125.1, 116.3, 103.4, 72.5, 60.1, 58.0, 14.3. FTIR (KBr): 3200–3032 (OH + NH), 1719 (COOEt), 1692 (CONH) cm^−1^. Anal. (C_22_H_21_N_4_O_4_SF) calcd for C, H, N, S (see Table 7).

*Ethyl 5-(3-(3-fluorobenzoyl)-thioureido)-1-(2-hydroxy-2-phenylethyl)-1H-pyrazole-4-carboxylate***4b**. White solid. Yield 48%; mp 155–156 °C. ^1^H-NMR (400 MHz, DMSO-d_6_): δ 1.13 (t, *J* = 7.0, 3H, CH_3_), 4.06–4.17 (m, 4H, CH_2_O + CH_2_N), 4.91–4.94 (m, 1H, *CH*OH), 5.78 (br s, 1H, OH, exchangeable), 7.21–7.85 (m, 9H, Ar), 7.89 (s, 1H, H-3). ^13^C NMR (101 MHz, DMSO-d_6_): δ 177.0, 164.4, 161.3, 159.2, 142.3, 140.2, 132.3, 130.0, 128.4, 127.2, 125.1, 116.4, 103.5, 72.6, 60.2, 57.9, 14.3. FTIR (KBr): 3350–3050 (OH + NH), 1708 (COOEt), 1677 (CONH) cm^−1^. Anal. (C_22_H_21_N_4_O_4_SF) calcd for C, H, N, S (see Table 7). 

*Ethyl 5-(3-(4-fluorobenzoyl)-thioureido)-1-(2-hydroxy-2-phenylethyl)-1H-pyrazole-4-carboxylate***4c**. White solid. Yield 51%; mp 158–160 °C. ^1^H-NMR (400 MHz, DMSO-d_6_): δ 1.22 (t, *J* = 7.0, 3H, CH_3_), 3.95–4.15 (m, 4H, CH_2_O + CH_2_N), 4.89–4.94 (m, 1H, *CH*OH), 5.94 (br s, 1H, exchangeable), 7.21–7.45 (m, 9H Ar), 7.88 (s, 1H, H-3), 8.07 (br s, 1H, exchangeable), 12.03 (br s, 1H, exchangeable). ^13^C NMR (101 MHz, CDCl_3_): δ 177.0, 164.6, 161.6, 159.3, 142.4, 140.3, 132.4, 130.1, 128.6, 127.2, 125.1, 116.5, 103.7, 72.7, 60.1, 57.2, 14.4. FTIR (KBr): 3281–2900 (OH + NH), 1711 (COOEt), 1676 (CONH) cm^−1^. Anal. (C_22_H_21_N_4_O_4_SF) calcd for C, H, N, S (see Table 7). 

#### 3.2.2. Synthesis of Compounds **5a-d**

To a mixture of 5-amino-pyrazole **1a** [17] (1.37 g, 5 mmol) in anhydrous THF (10 mL), TEA (1 mL) and the suitable benzoyl chloride (6 mmol) were slowly added at 0 °C; then, the mixture was refluxed for 18 h. After cooling to room temperature, the solvent was removed under reduced pressure and the crude was solved in DCM (15 mL). The organic phase was washed with 4M NaOH (150 mL), 3M HCl (15 mL), water (15 mL), dried (MgSO_4_) and concentrated under reduced pressure. The crude crystallized by adding a solution of diethyl ether/petroleum ether (b.p. 50–60 °C) (1:1).

*Ethyl 5-benzamido-1-(2-hydroxy-2-phenylethyl)-1H-pyrazole-4-carboxylate **5a***. White solid. Yield 61%; mp 117–118 °C. ^1^H-NMR (200 MHz, CDCl_3_): δ 1.25 (t, *J* = 7.0, 3H, CH_3_), 4.30 (q, J = 7.0, 2H, CH_2_O), 4.35–4.90 (2m, 2H, CH_2_N), 6.29–6.47 (m, 1H, *CH*OH), 6.70 (br s, 1H, exchangeable), 7.53–8.25 (m, 11H, 10Ar + H-3). ^13^C NMR (101 MHz, CDCl_3_): δ 177.0, 164.6, 161.4, 159.2, 142.3, 140.2, 132.2, 130.0, 128.4, 127.2, 125.1, 116.3, 103.4, 72.5, 60.1, 58.0, 14.3. FTIR (KBr): 3461, 3360 (OH + NH), 1681 (COOEt), 1697 (CONH) cm^−1^. Anal. (C_21_H_21_N_3_O_4_) calcd for C, H, N (see Table 7).

*Ethyl 5-(3-fluorobenzamido)-1-(2-hydroxy-2-phenylethyl)-1H-pyrazole-4-carboxylate **5b***. White solid. Yield 35%; mp 149–151 °C. ^1^H-NMR (200 MHz, CDCl_3_): δ 1.49 (t, *J* = 7.0, 3H, CH_3_), 4.43 (q, J = 7.0, 2H, CH_2_O), 4.90–5.30 (m, 2H, CH_2_N), 6.42 (t, 1H, OH exchangeable), 6.58–6.67 (m, 1H, *CH*OH), 7.35–8.15 (m, 10H, 9Ar + H-3), 9.43 (br s, 1H, NH exchangeable). ^13^C NMR (101 MHz,CDCl_3_): δ 166.2, 163.9, 162.0, 142.3, 141.6, 140.2, 135.6, 130.6, 128.4, 128.0, 127.2, 123.5, 119.0, 114.3, 102.4, 72.5, 60.1, 58.0, 14.3. FTIR (KBr): 3200–2974 (OH + NH), 1732 (COOEt), 1710 (CONH) cm^−1^. Anal. (C_21_H_20_N_3_O_4_F) calcd for C, H, N (see Table 7).

*Ethyl 5-(4-fluorobenzamido)-1-(2-hydroxy-2-phenylethyl)-1H-pyrazole-4-carboxylate **5c**.* White solid. Yield 30%; mp 98–100 °C. ^1^H-NMR (400 MHz, DMSO-d_6_): δ 1.16 (t, *J* = 7.0, 3H, CH_3_), 4.10 (q, J = 7.0, 2H, CH_2_O), 4.21–4.64 (2m, 2H, CH_2_N), 4.94 (br s, 1H, OH exchangeable), 6.09–6.20 (m, 1H, *CH*OH), 6.50 (br s, 1H, OH exchangeable), 7.20–7.42 (m, 7H, Ar), 7.51 (s, 1H, H-3), 8.21–8.28 (m, 2H, Ar). ^13^C NMR (101 MHz, CDCl_3_): δ 165.9, 163.8, 161.4, 142.3, 141.6, 140.2, 130.2, 130.2, 129.8, 129.8, 128.4, 128.0, 127.2, 116.0, 115.9, 102.4, 72.5, 60.1, 58.0, 14.3. FTIR (KBr): 3408, 3292, 3226 (OH + NH), 1708 (COOEt), 1684 (CONH) cm^−1^. Anal. (C_21_H_20_N_3_O_4_F) calcd for C, H, N (see Table 7).

*Ethyl 1-(2-hydroxy-2-phenylethyl)-5-(3-(trifluoromethyl)-benzamido)-1H-pyrazole-4-carboxylate **5d**.* Yellow oil. Yield 23%. ^1^H-NMR (400 MHz, CDCl_3_): δ 1.15 (t, *J* = 7.0, 3H, CH_3_), 4.07 (q, J = 7.0, 2H, CH_2_O), 4.25–4.69 (2m, 2H, CH_2_N), 6.19–6.52 (m, 1H, *CH*OH), 6.52 (br s, 1H, exchangeable), 7.23–8.30 (m, 10H, 9Ar + H-3). ^13^C NMR (101 MHz, CDCl_3_): 166.3, 161.4, 142.3, 141.6, 140.2, 133.7, 131.6, 130.8, 129.0, 129.0, 127.2, 126.9, 124.9, 124.6, 122.7, 120.5, 102.4, 72.5, 60.1, 58.0, 14.3. FTIR (KBr): FTIR (KBr): 3450, 3346 (OH + NH), 1727 (COOEt), 1683 (CONH) cm^−1^. Anal. (C_21_H_20_N_3_O_4_F) calcd for C, H, N (see Table 7).

#### 3.2.3. Synthesis of Ethyl 5-((4-fluorophenyl)-sulfonamido)-1-(2-hydroxy-2-phenylethyl)-1H-pyrazole-4-carboxylate **6**

To a mixture of 5-amino-pyrazole **1a** [17] (1.37 g, 5 mmol) in anhydrous THF (10 mL), TEA (1 mL) and the 4-fluorobenzenesulfonyl chloride (1.17 g, 6 mmol) were slowly added at 0 °C; then, the mixture was refluxed for 3 days. After cooling to room temperature, the solvent was removed under reduced pressure and the crude was solved in DCM (15 mL). The organic phase was washed with 4M NaOH (15 mL), 3 M HCl (15 mL), water (15 mL), dried (MgSO_4_) and concentrated under reduced pressure. The crude crystallized by adding a solution of diethyl ether/petroleum ether (b.p. 50–60 °C) (1:1). The white solid obtained was recrystallized from absolute ethanol. Yield 34%; mp 180–181 °C. ^1^H-NMR (400 MHz, DMSO-d_6_): δ 1.19 (t, *J* = 7.0, 3H, CH_3_), 3.94–4.16 (m, 4H, CH_2_O + CH_2_N), 4.90–4.93 (m, 1H, *CH*OH), 6.90 (br s, 1H, exchangeable), 7.07–7.30 (m, 8H, Ar), 7.55 (s, 1H, H-3), 7.58–7.61 (m, 1H, Ar). ^13^C NMR (101 MHz, CDCl_3_): δ 161.2, 158.1, 156.2, 143.7, 142.3, 140.0, 134.7, 131.3, 131.2, 128.4, 128.0, 127.1, 126.3, 126.1, 104.7, 72.5, 60.1, 57.8, 14.2. FTIR (KBr): 3399, 3303, 3177 (OH, NH), 1698 (COOEt) cm^−1^. Anal. (C_20_H_20_N_3_O_5_SF) calcd for C, H, N, S (see Table 7).

#### 3.2.4. Synthesis of Ethyl 5-(3-(4-fluorobenzoyl)-thioureido)-1-(2-hydroxy-2-phenylethyl)-1H-pyrazole-3-carboxylate **7**

A mixture of 5-amino-pyrazole **1b** [11] (0.275 g, 1 mmol) and the 4-fluorobenzoyl isothiocyanate (0.181 g, 1 mmol), previously prepared following a method from the literature [18], in anhydrous THF (10 mL) was refluxed for 12 h. After cooling to room temperature, the white solid obtained was filtered and purified by flash chromatography using diethyl ether as the eluent. White solid. Yield 37%; mp 169–172 °C. ^1^H-NMR (400 MHz, DMSO-d_6_): δ 1.27 (t, *J* = 7.0, 3H, CH_3_), 4.13–4.30 (m, 4H, CH_2_O + CH_2_N), 4.86–4.91 (m, 1H, *CH*OH), 5.92 (br s, 1H, exchangeable), 7.07 (br s, 1H, exchangeable), 7.23–7.57 (m, 8H 7Ar + H-3), 8.03–8.09 (m, 2H Ar), 12.00 (br s, 1H, exchangeable). ^13^C NMR (101 MHz, CDCl_3_): δ 175.6, 165.8, 165.8, 163.8, 162.1, 143.3, 142.3, 141.2, 131.5, 131.5, 130.2, 130.1, 128.4, 128.0, 127.2, 116.0, 115.8, 100.6, 72.5, 60.9, 57.7, 14.3. FTIR (KBr): 3450, 3350, 3069 (OH + NH), 1712 (COOEt) cm^−1^. Anal. (C_22_H_21_N_4_O_4_SF) calcd for C, H, N, S (see Table 7).

### 3.3. Biological Evaluation

#### 3.3.1. Microbiology

##### Bacterial Species Considered in This Study

Since in preliminary experiments none of the compounds reported in this study were active on bacteria of the Gram-negative species, in the following experiments several clinical isolates of Gram-positive species were tested for a total of 21 strains of *Enterococcus* and *Staphylococcus* genus. All bacteria belonged to a collection obtained from the School of Medicine and Pharmacy of University of Genoa (Italy). Their identification was carried out as described in our previous works [24,25]. Particularly, 9 strains were of the *Enterococcus* genus, including 3 vancomycin-resistant (VRE) *E. faecalis* strains, 3 VRE *E. faecium* isolates, 1 VRE *E. durans,* 1 VRE *E. gallinarum* and 1 VRE *E. casseliflavus.* The other clinical isolates were staphylococci. Particularly, 3 isolates were methicillin-resistant *S. aureus* (MRSA), 3 were methicillin-resistant *S. epidermidis* (MRSE), while 6 were strains belongonging to other species of the genus *Stafilococcus,* of which 3 resistant to methicillin (1 *S. hominis*, 1 *S. simula*ns and 1 *S. haemolyticus*) and 3 susceptible to methicillin (including 1 *S. saprophyticus*, 1 *S. warneri* and 1 *S. lugdunensis*).

##### Determination of the Minimal Inhibitory Concentrations (MICs)

To assess the antimicrobial activity of compounds **3a**–**c** and **4a**–**c** their MICs were determined following the microdilution procedures detailed by the European Committee on Antimicrobial Susceptibility Testing EUCAST [26], as also reported in our previous studies [24,25]. Here, serial 2-fold dilutions of solutions of all the six samples (DMSO), ranging from 1 to 256 µg/mL, were used, using DMSO as control to verify the absence of antibacterial activity of the solvent used for the experiments. All MICs were obtained at least in triplicate, and results were expressed reporting the modal value, that is the value that has been observed most frequently. In the case of equivocal or unclear results, more than three determinations of MICs were carried out.

#### 3.3.2. MABA and LORA Tests

MICs against replicating and non-replicating cultures of *M. tuberculosis* were determined using the Microplate Alamar Blue Assay (MABA) and the Low Oxygen Recovery Assay (LORA) [22].

## 4. Conclusions

To further extend the SARs of **BBB4** and **CB1H**, **5-PUs** and **I** derivatives, the synthesis, characterization, and microbiological evaluation of a small library of trisubstituted pyrazoles have been here reported. In addition, the compounds demonstrating more promise for future development of new drug delivery systems by nanoencapsulation have been found. Structurally, compounds **2**–**7** shared the N1 2-hydroxy-2-phenylethyl chain, previously identified as a relevant structural determinant for biological activity [8,9,10,11,12,13]. The compounds were prepared by a divergent synthetic protocol starting from the key intermediates **1a**–**c** and were tested on a panel of twenty-one MDR clinical isolates of the *Staphylococcus* and *Enterococcus* genus. Compounds **3c** and **4b**, characterized by a urea or thiourea function at C5 of the pyrazole nucleus, respectively, were found to be the most active derivatives as antibacterial agents, whereas derivatives **3c** and **4a** show significant, albeit moderate, activity against *M. tuberculosis,* with **4a** being weakly active against an MDR TB strain. Collectively, the presence of an amide (compounds **5**) or sulfonamide (compounds **6**) moiety at the C5 position negatively affected the biological properties. In silico study predicted these compounds to exert good drug-like and pharmacokinetic properties.

Overall, the collected data support the pharmaceutical potentials of derivatives **3** and **4** as novel antibacterial agents. In fact, the biological results, particularly against clinical isolates of enterococci and staphylococci, confirm that the N1 2-hydroxy-2-phenylethyl chain induces an improvement in antimicrobial activity with respect to previously reported **BBB4** and **CB1H**, which were completely inactive when not encapsulated in proper nanosized macromolecules. Based on the excellent antibacterial effects obtained encapsulating the inactive **BBB4** and **CB1H** in NPs, pyrazoles **3c** and **4b** here reported represent even more promising candidates for future development of pyrazole-based nano-delivery systems with further enhanced antibacterial properties in terms of potency and spectrum of activity. 

In conclusion, the pyrazole-based library here developed contains at least two compounds, namely **3c** and **4b** which, upon nano-formulation with proper polymer matrices, could provide new pyrazole-based drug delivery systems with enhanced and broader-spectrum antibacterial activity.

## Figures and Tables

**Table 1:**
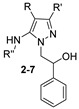
Chemical structure, melting point and reaction yield of new pyrazole derivatives **2**–**7**.

Compd.	R	R’	R’’	M.p (°C)	Yield (%)
**2a**	COOEt	H	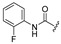	[11]	[11]
**2b**	COOEt	H	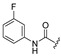	[11]	[11]
**2c**	COOEt	H	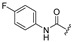	[11]	[11]
**2d**	COOEt	H	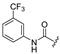	[11]	[11]
**3a**	H	C(CH_3_)_3_	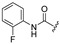	[12]	[12]
**3b**	H	C(CH_3_)_3_	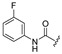	[12]	[12]
**3c**	H	C(CH_3_)_3_	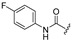	[12]	[12]
**3d**	H	C(CH_3_)_3_	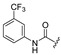	[12]	[12]
**4a**	COOEt	H	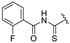	150–151	42
**4b**	COOEt	H	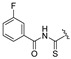	155–156	48
**4c**	COOEt	H	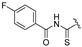	158–160	51
**5a**	COOEt	H	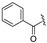	117–118	61
**5b**	COOEt	H	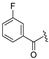	149–151	35
**5c**	COOEt	H	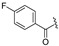	98–100	30
**5d**	COOEt	H	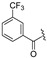	Oil	23
**6**	COOEt	H	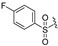	180–181	34
**7**	H	COOEt	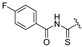	169–172	37

**Table 2:** Predicted pharmacokinetics and drug-like properties of compounds **2a**–**d** and **3a**–**d**.

	2a	2b	2c	2d	3a	3b	3c	3d
**Physicochemical Prop.**								
MW (g/mol)	412.41	412.41	412.41	462.42	396.46	396.46	396.46	446.47
Fraction Csp^3^	0.19	0.19	0.19	0.23	0.27	0.27	0.27	0.3
Rotatable bonds	10	10	10	11	8	8	8	9
H-bond acceptors	6	6	6	8	4	4	4	6
H-bond donors	3	3	3	3	3	3	3	3
TPSA ^a^ (Å^2^)	105.48	105.48	105.48	105.48	79.18	79.18	79.18	79.18
**Lipophilicity**								
LogP ^b^	2.78	2.78	2.78	3.56	3.72	3.72	3.72	4.5
**Water solubility**								
Solubility (mg/mL) ^c^	0.051	0.051	0.051	0.0115	0.0112	0.0112	0.0112	0.00256
Solubility class	Soluble	Soluble	Soluble	Moderately soluble	Moderately soluble	Moderately soluble	Moderately soluble	Moderately soluble
**Pharmacokinetics**								
GI absorption	High	High	High	High	High	High	High	High
BBB permeant	No	No	No	No	No	No	No	No
Pgp substrate	Yes	Yes	Yes	No	No	No	Yes	No
CYP1A2 inhibitor	No	No	No	No	No	No	No	No
CYP2C19 inhibitor	No	Yes	Yes	Yes	Yes	Yes	Yes	Yes
CYP2C9 inhibitor	Yes	Yes	Yes	Yes	Yes	Yes	Yes	No
CYP2D6 inhibitor	No	No	No	No	No	No	No	No
CYP3A4 inhibitor	No	No	No	Yes	Yes	Yes	Yes	Yes
**Druglikeness**								
Lipinski violations	0	0	0	0	0	0	0	0
**Medicinal chemistry**								
PAINS alerts	0	0	0	0	0	0	0	0
Brenk alerts	0	0	0	0	0	0	0	0

^a^ Topological Polar Surface Area. ^b^ Predicted according to XLOGP3 program. ^c^ Values predicted by ESOL method [21].

**Table 3:** Predicted pharmacokinetics and drug-like properties of compounds **4**–**7**.

	4a	4b	4c	5a	5b	5c	5d	6	7
**Physicochemical Properties**									
MW (g/mol)	456.49	456.49	456.49	379.41	397.40	397.40	447.41	433.45	456.49
Fraction Csp^3^	0.18	0.18	0.18	0.19	0.19	0.19	0.23	0.20	0.18
Rotatable bonds	11	11	11	9	9	9	10	9	11
H-bond acceptors	6	6	6	5	6	6	8	7	6
H-bond donors	3	3	3	2	2	2	2	2	3
TPSA ^a^ (Å^2^)	137.57	137.57	137.57	93.45	93.45	93.45	93.45	118.9	137.57
**Lipophilicity**									
LogP ^b^	3.26	3.26	3.26	2.95	3.05	3.05	3.83	2.74	3.59
**Water solubility**									
Solubility (mg/mL) ^c^	0.0185	0.0185	0.0185	0.0471	0.0342	0.0342	0.00778	0.0361	0.0115
Solubility class	Moderately soluble	Moderately soluble	Moderately soluble	Soluble	Moderately soluble	Moderately soluble	Moderately soluble	Moderately soluble	Moderately soluble
**Pharmacokinetics**									
GI absorption	Low	Low	Low	High	High	High	High	High	Low
BBB permeant	No	No	No	No	No	No	No	No	No
Pgp substrate	No	No	No	No	No	No	No	No	No
CYP1A2 inhibitor	No	No	No	No	Yes	Yes	No	No	No
CYP2C19 inhibitor	Yes	Yes	Yes	Yes	Yes	Yes	Yes	Yes	Yes
CYP2C9 inhibitor	Yes	Yes	Yes	Yes	Yes	Yes	Yes	Yes	Yes
CYP2D6 inhibitor	Yes	No	Yes	Yes	Yes	Yes	Yes	No	No
CYP3A4 inhibitor	Yes	Yes	Yes	No	No	No	Yes	Yes	Yes
**Druglikeness**									
Lipinski violations	0	0	0	0	0	0	0	0	0
**Medicinal chemistry**									
PAINS alerts	0	0	0	0	0	0	0	0	0
Brenk alerts	1	1	1	0	0	0	0	0	1

^a^ Topological Polar Surface Area. ^b^ Predicted according to XLOGP3 program. ^c^ Values predicted by ESOL method [21].

**Table 4:** MIC values of pyrazoles **3a**–**c** and **4a**–**c** against bacteria of the Gram-positive species, obtained from experiments carried out at least in triplicate, expressed as µg/mL.

Strains	MIC (µg/mL)					
	3a	3b	3c	4a	4b	4c
***Enterococcus genus***						
*E. faecalis* 365 *	>128	>128	128	>128	>128	>128
*E. faecalis* 450 *	128	128	**64**	>128	>128	>128
*E. faecalis* 451 *	>128	128	**64**	>128	>128	>128
*E. faecium* 182 *	128	**64**	**64**	>128	>128	>128
*E. faecium* 300 *	>128	>128	**64**	>128	>128	>128
*E. faecium* 364 *	>128	>128	**64**	>128	>128	>128
*E. durans* 103	**64**	>128	**64**	>128	>128	>128
*E. gallinarum* 150 *	**32**	>128	128	>128	>128	>128
*E. casseliflavus* 184	**64**	**64**	128	128	>128	>128
***Staphylococcus genus***						
*S. aureus* 18 **	>128	>128	**64**	>128	**64**	128
*S. aureus* 187 **	>128	128	**32**	128	**64**	**64**
*S. aureus* 195 **	128	128	**32**	>128	128	128
*S. epidermidis* 180 ***	128	64	**32**	128	**64**	128
*S. epidermidis* 181 ***	>128	128	**64**	128	**64**	128
*S. epidermidis* 363 **	128	128	**32**	128	128	128
*S. saprophyticus* 41	>128	>128	**64**	>128	>128	>128
*S. warneri* 74	**64**	128	**64**	128	>128	>128
*S. hominis* 125 #	**32**	**32**	**64**	128	128	128
*S. lugdunensis* 129	**32**	**32**	**32**	**64**	**32**	**32**
*S. simulans* 163 #	>128	>128	128	>128	>128	>128
*S. haemolyticus* 193 #	>128	>128	128	>128	>128	>128

* Denotes vancomycin resistant (VRE), ** denotes methicillin resistant; *** denotes resistance toward methicillin and linezolid; # denotes methicillin resistant; bold data evidence promising antibacterial activity.

**Table 5:** MIC values of compounds **3c** and **4b** against the most clinically relevant bacteria of the Gram-positive species, obtained from experiments carried out at least in triplicate, and those of reference antibiotics expressed as µg/mL.

	MIC (µg/mL)				
Strains	3c	4b	Ampicillin	Ciprofloxacin	Oxacillin
*E. faecalis* 365 *	128	>128	128	-	-
*E. faecalis* 450 *	**64**	>128	128	-	-
*E. faecalis* 451 *	**64**	>128	128	-	-
*E. faecium* 182 *	**64**	>128	128	-	-
*E. faecium* 300 *	**64**	>128	128	-	-
*E. faecium* 364 *	**64**	>128	128	-	-
*S. aureus* 18 **	**64**	**64**	-	128	512
*S. aureus* 187 **	**32**	**64**	-	128	512
*S. aureus* 195 **	**32**	128	-	128	512
*S. epidermidis* 180 ***	**32**	**64**	-	**64**	256
*S. epidermidis* 181 ***	**64**	**64**	-	**64**	256
*S. epidermidis* 363 **	**32**	**64**	-	**64**	256

* Denotes vancomycin resistant (VRE); ** denotes methicillin resistant; *** denotes resistance toward methicillin and linezolid; bold data evidence promising antibacterial activity.

**Table 6:** Percentage of inhibition at 50 μg/mL concentration and MIC values (µg/mL) in the MABA test for compounds **3a**–**d** and **4a**–**c**. These values are the result of the average of two independent experiments; bold data evidence promising antibacterial activity.

Entry	% inhib. at 50 µg/mL	MIC (µg/mL)
**3a**	30	>50
**3b**	55	>50
**3c**	99	**35.8**
**3d**	59	>50
**4a**	100	**39.3**
**4b**	75	>50
**4c**	88	>50

**Table 7:** Elemental analysis of compounds **4**-**7**.

Compd.	Molecular Formula	Elemental Analysis				%S
		Values	%C	%H	%N	
**4a**	C_22_H_21_N_4_O4SF	Calcd.	57.88	4.64	12.27	7.02
		Found	57.77	4.50	12.29	6.76
**4b**	C_22_H_21_N_4_O4SF	Calcd.	57.88	4.64	12.27	7.02
		Found	57.87	4.50	12.36	6.83
**4c**	C_22_H_21_N_4_O4SF	Calcd.	57.88	4.64	12.27	7.02
		Found	57.57	4.91	12.40	7.24
**5a**	C_21_H_21_N_3_O_4_	Calcd.	66.48	5.58	11.08	//
		Found	66.16	5.80	11.09	//
**5b**	C_21_H_20_N_3_O_4_F	Calcd.	63.47	5.07	10.57	//
		Found	63.35	4.87	10.46	//
**5c**	C_21_H_20_N_3_O_4_F	Calcd.	63.47	5.07	10.57	//
		Found	63.47	5.32	10.57	//
**5d**	C_22_H_20_N_3_O_4_F_3_	Calcd.	59.06	4.51	9.39	//
		Found	59.00	4.60	9.20	//
**6**	C_20_H_20_N_3_O5SF	Calcd.	55.42	4.65	9.69	7.40
		Found	55.88	4.77	9.41	7.14
**7**	C_22_H_21_N_4_O4SF	Calcd.	57.88	4.64	12.27	7.02
		Found	57.92	4.36	12.27	7.15

## Data Availability

Not applicable.

## Supplementary Materials

The following supporting information can be downloaded at: https://www.mdpi.com/article/10.3390/pharmaceutics14091770/s1. Figure S1. ^1^H NMR of 4c. Figure S2. ^13^C NMR of 4c. Figure S3. IR of 4c. Figure S4. ^1^H NMR of 5c. Figure S5. ^13^C NMR of 5c. Figure S6. IR of 5c. Figure S7. ^1^H NMR of 5d. Figure S8. ^13^C NMR of 5d. Figure S9. IR of 5d. Figure S10. ^1^H NMR of 6. Figure S11. ^13^C NMR of 6. Figure S12. IR of 6. Figure S13. ^1^H NMR of 7. Figure S14. ^13^C NMR of 7. Figure S15. IR of **7**.
