# Supplementary material for: Microbiological Screening of 5-Functionalized Pyrazoles for the Future Development of Optimized Pyrazole-Based Delivery Systems
Source: Pharmaceutics. 2022 Aug 24;14(9):1770. doi: 10.3390/pharmaceutics14091770 (PMC9503297; doi:10.3390/pharmaceutics14091770)

Microbiological Screening of 5-Functionalized Pyrazoles for the Future Development of Optimized Pyrazole-Based Delivery Systems

Chiara Brullo [1] *, Debora Caviglia [2] , Andrea Spallarossa [1], Silvana Alfei [3], Scott G. Franzblau [4], Bruno Tasso [1], Anna Maria Schito [2]

[1] Department of Pharmacy (DIFAR), Section of medicinal Chemistry, University of Genoa, Viale Benedetto Xv, 3, 16132 Genoa, Italy
[2] Department of Surgical Sciences and Integrated Diagnostics (DISC), University of Genoa, Viale Benedetto XV, 6, 16132 Genoa, Italy
[3] Department of Pharmacy (DIFAR), Section of Organic Chemistry, University of Genoa, Viale Cembrano, 16148 Genoa, Italy
[4] Institute for Tuberculosis Research, College of Pharmacy, University of Illinois at Chicago, 833 South Wood Street, Chicago, IL 60612, USA
* Correspondence: chiara.brullo@unige.it

Supporting Information:

Figure S1. ^{1}H NMR of **4c**.
Figure S2. ^{13}C NMR of **4c**.
Figure S3. IR of **4c**
Figure S4. ^{1}H NMR of **5c**.
Figure S5. ^{13}C NMR of **5c**.
Figure S6. IR of **5c.**
Figure S7. ^{1}H NMR of **5d**.
Figure S8. ^{13}C NMR of **5d**.
Figure S9. IR of **5d.**
Figure S10. ^{1}H NMR of **6**.
Figure S11. ^{13}C NMR of **6**.
Figure S12. IR of **6.**
Figure S13. ^{1}H NMR of **7**.
Figure S14. ^{13}C NMR of **7**.
Figure S15. IR of **7.**

Figure S1. ^{1}H NMR of **4c**.

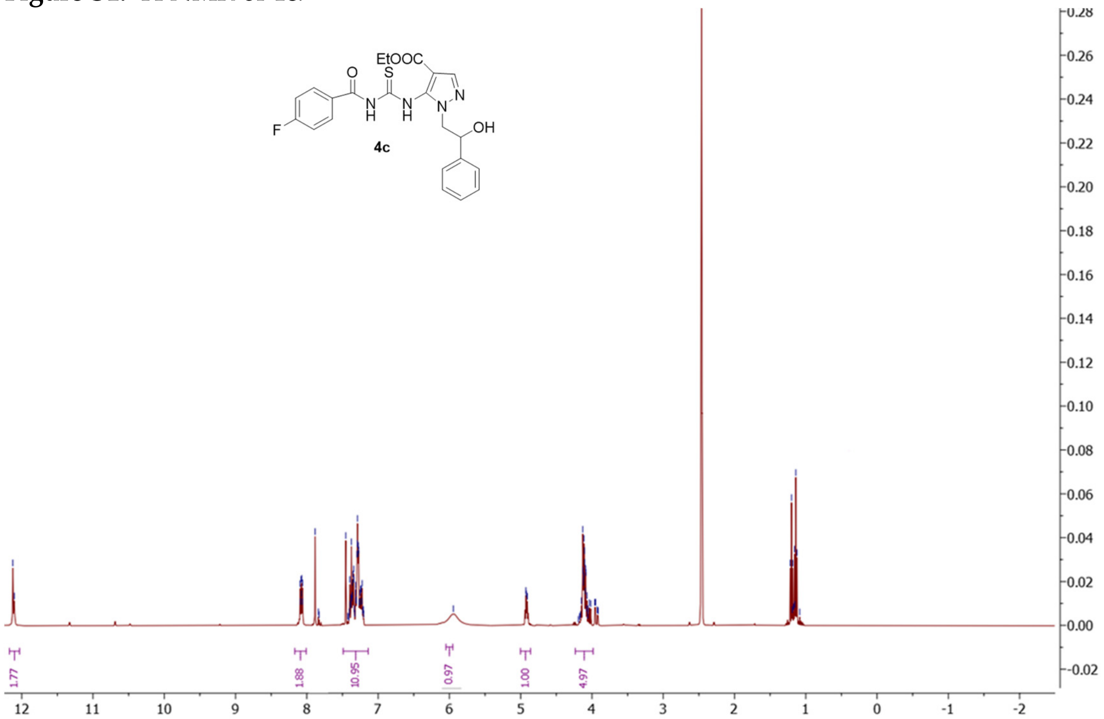

Figure S2. ^{13}C NMR of **4c**.

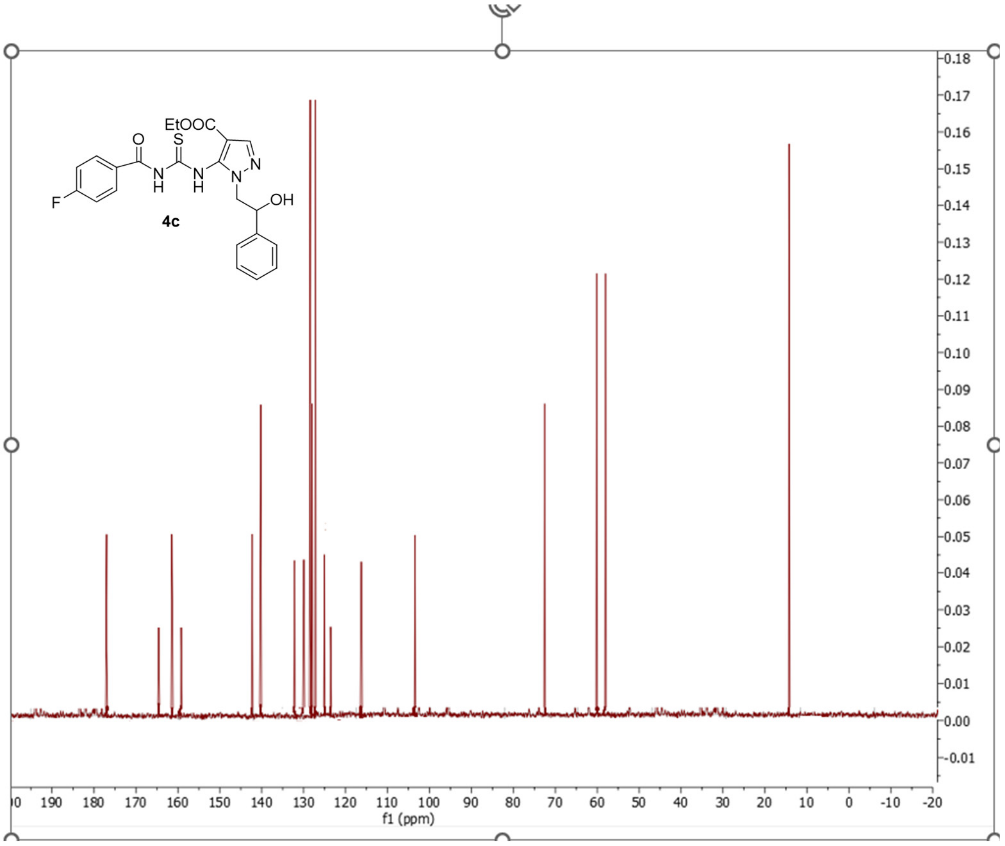

Figure S3. IR of **4c**

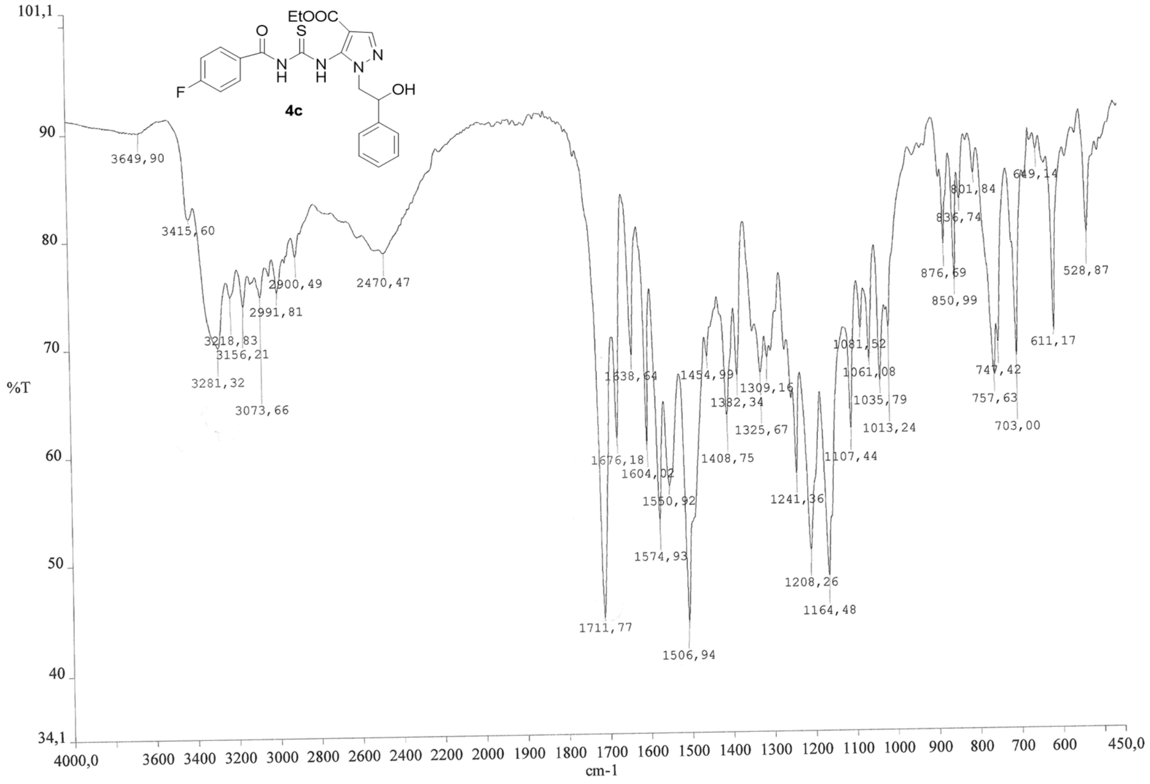

Figure S4. ^{1}H NMR of **5c**.

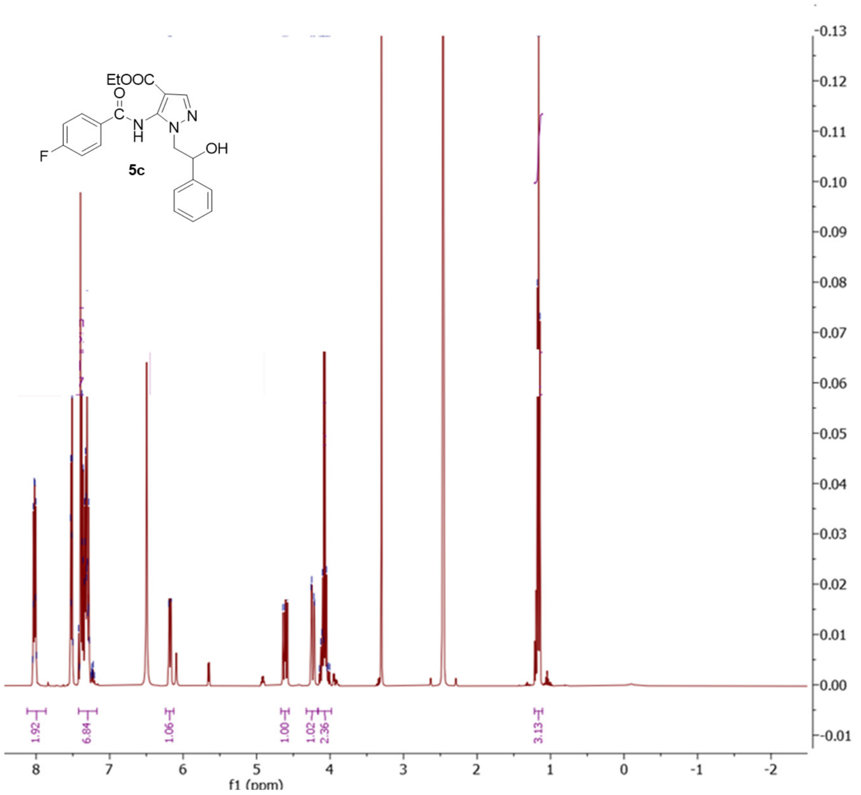

Figure S5. ^{13}C NMR of **5c**.

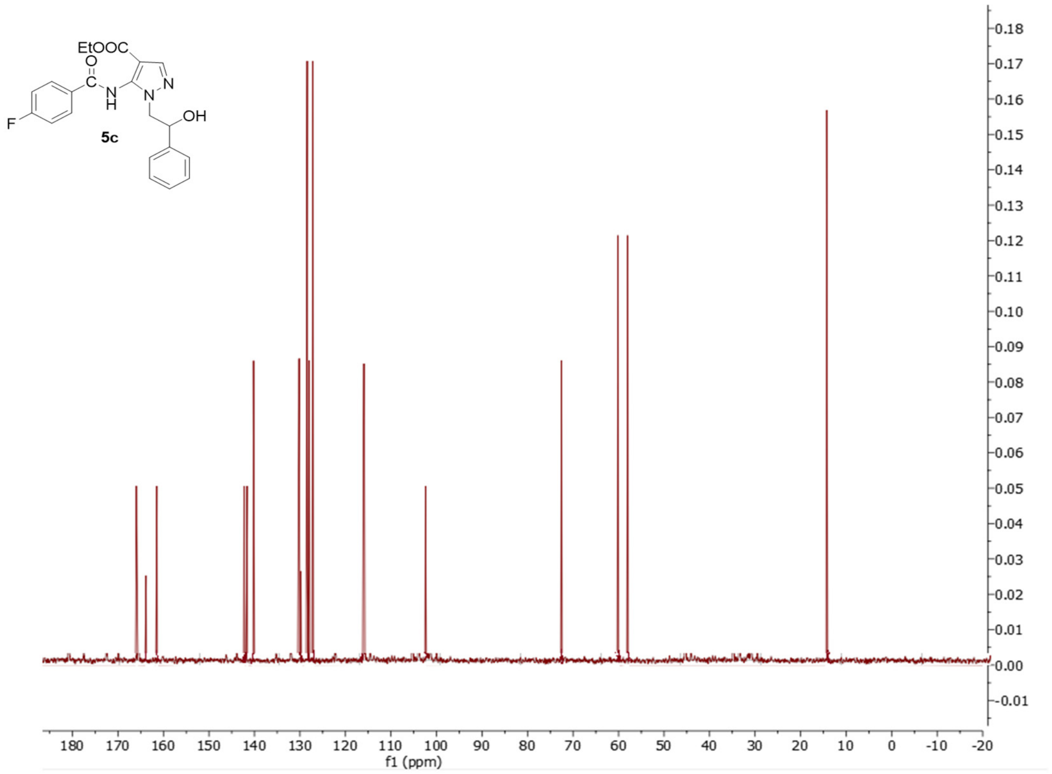

Figure S6. IR of **5c.**

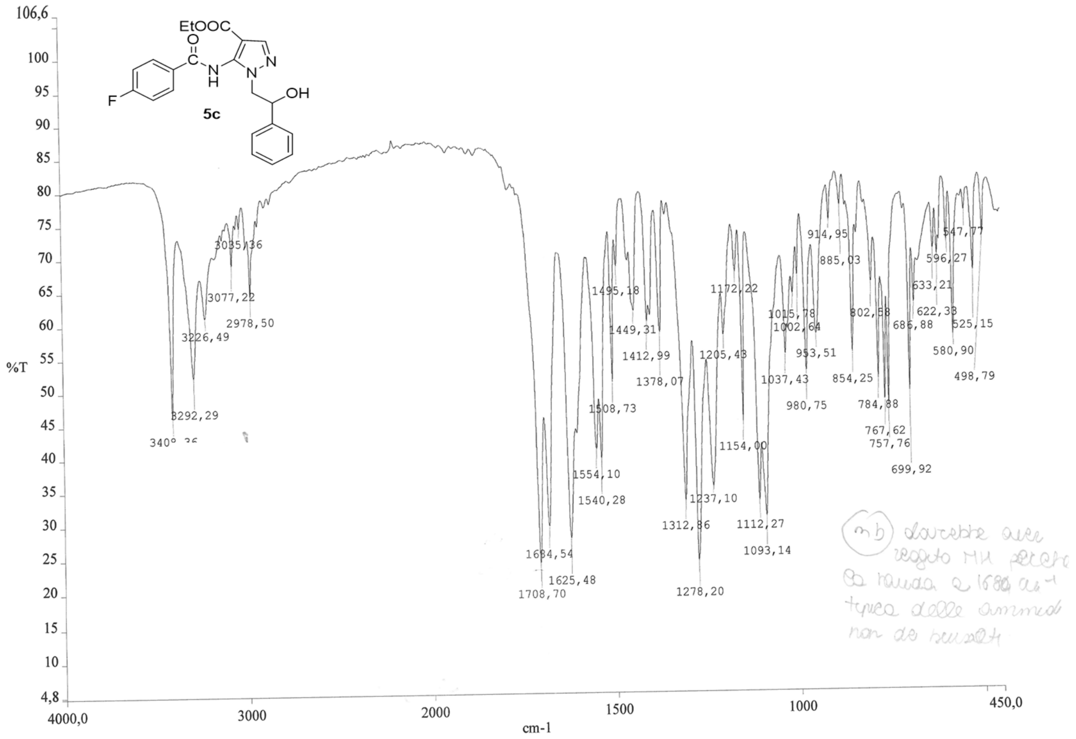

Figure S7. ^{1}H NMR of **5d**.

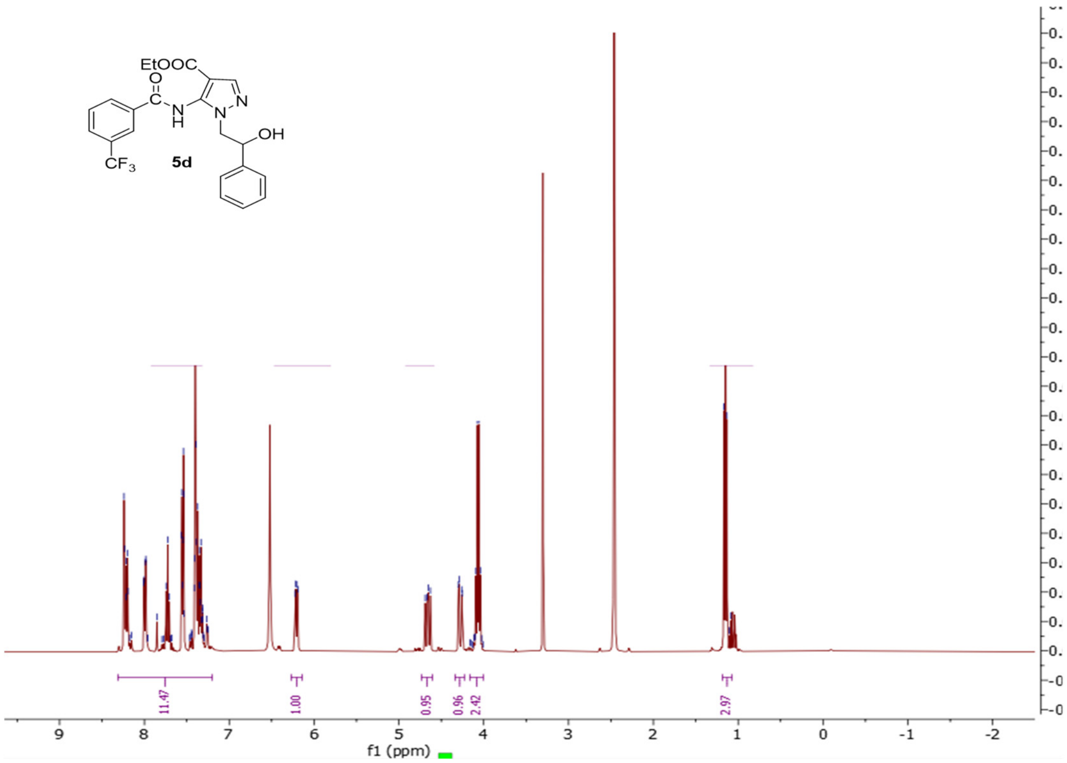

Figure S8. ^{13}C NMR of **5d**.

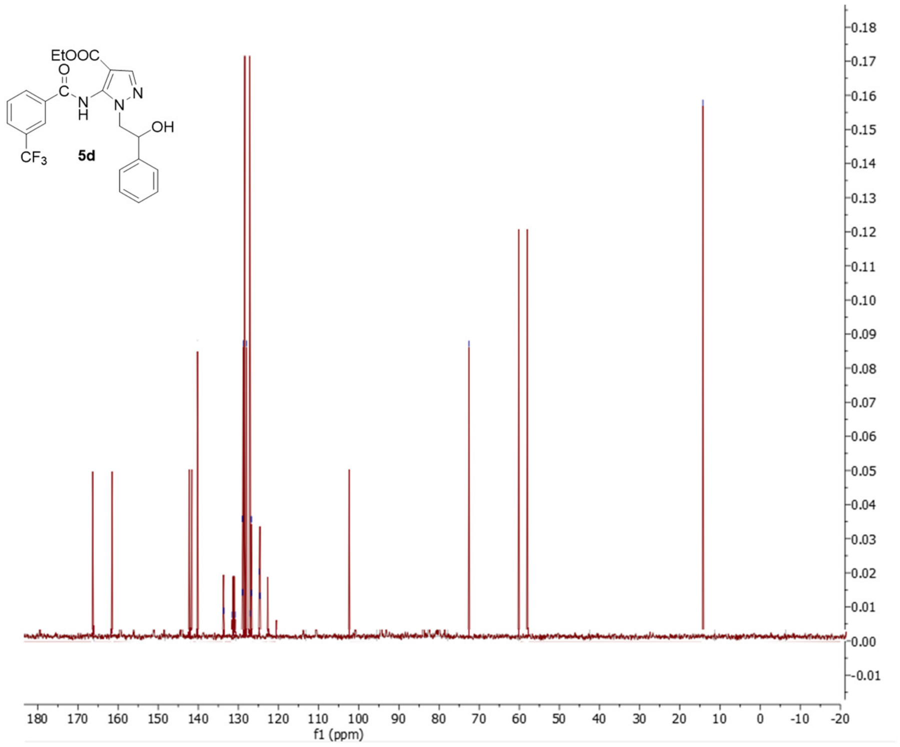

Figure S9. IR of **5d.**

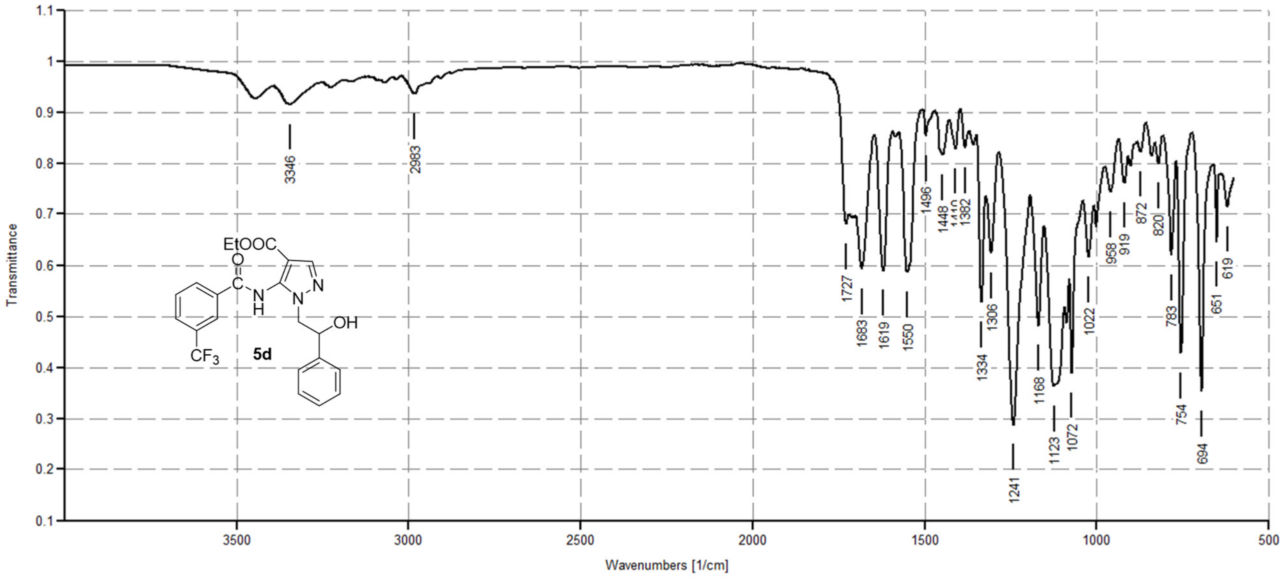

Figure S10. ^{1}H NMR of **6**.

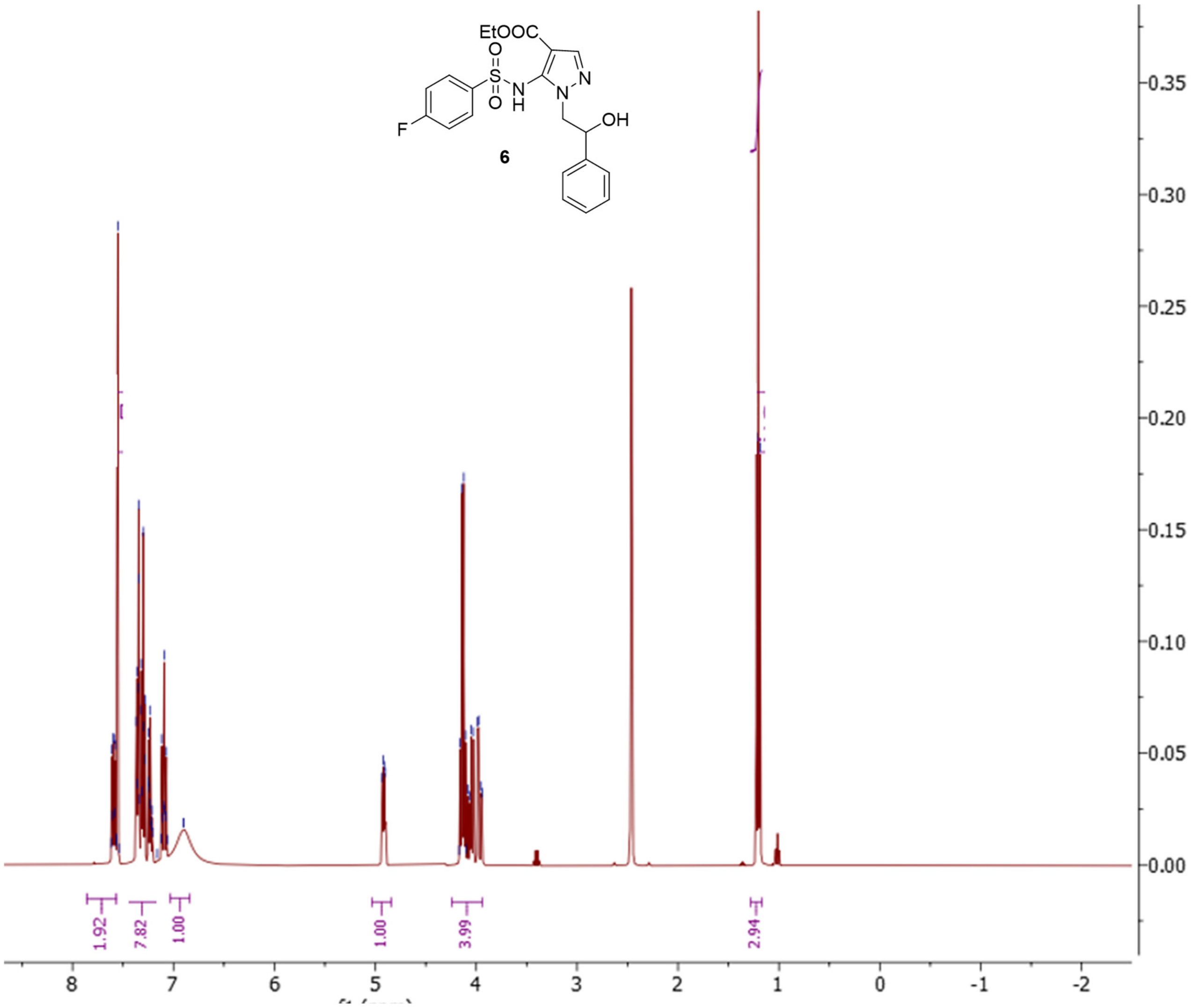

Figure S11. ^{13}C NMR of **6**.

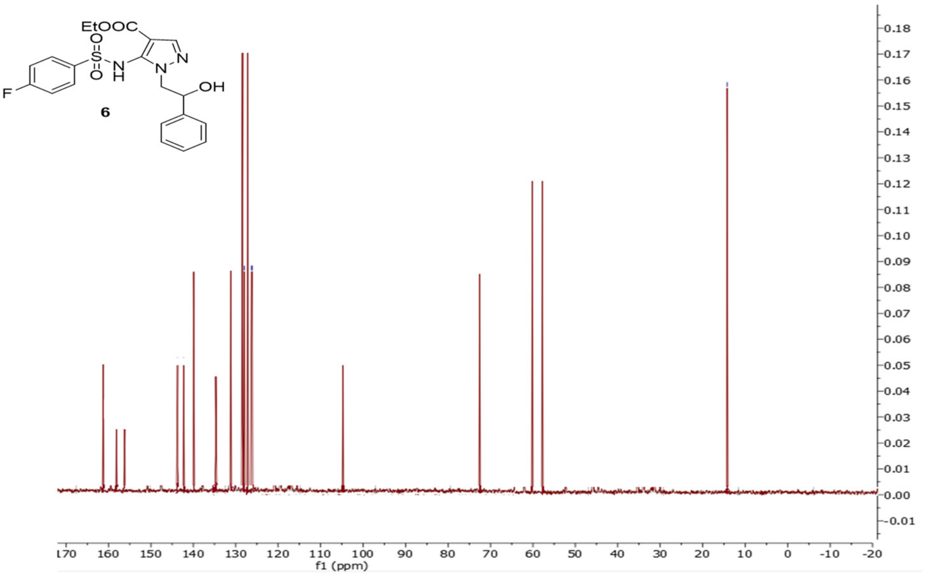

Figure S12. IR of **6.**

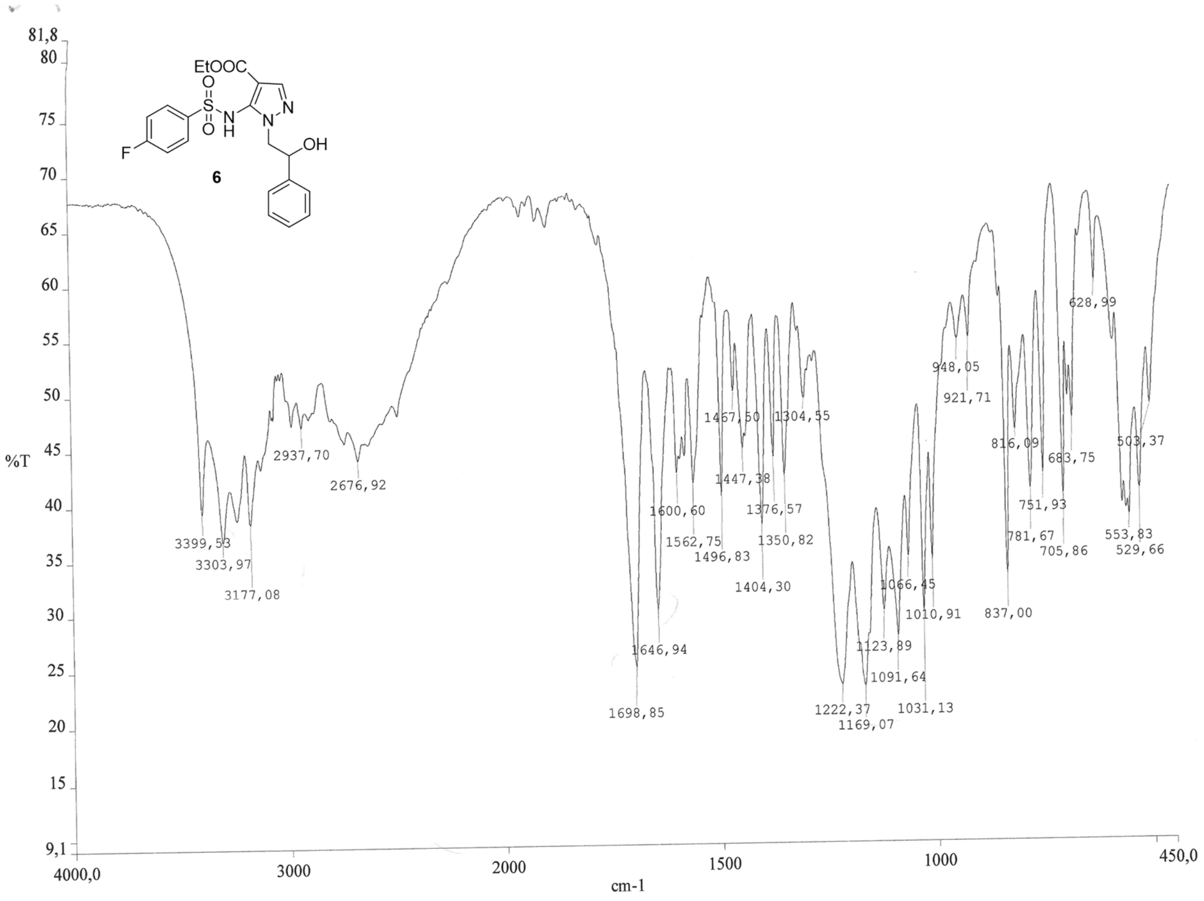

Figure S13. ^{1}H NMR of **7**.

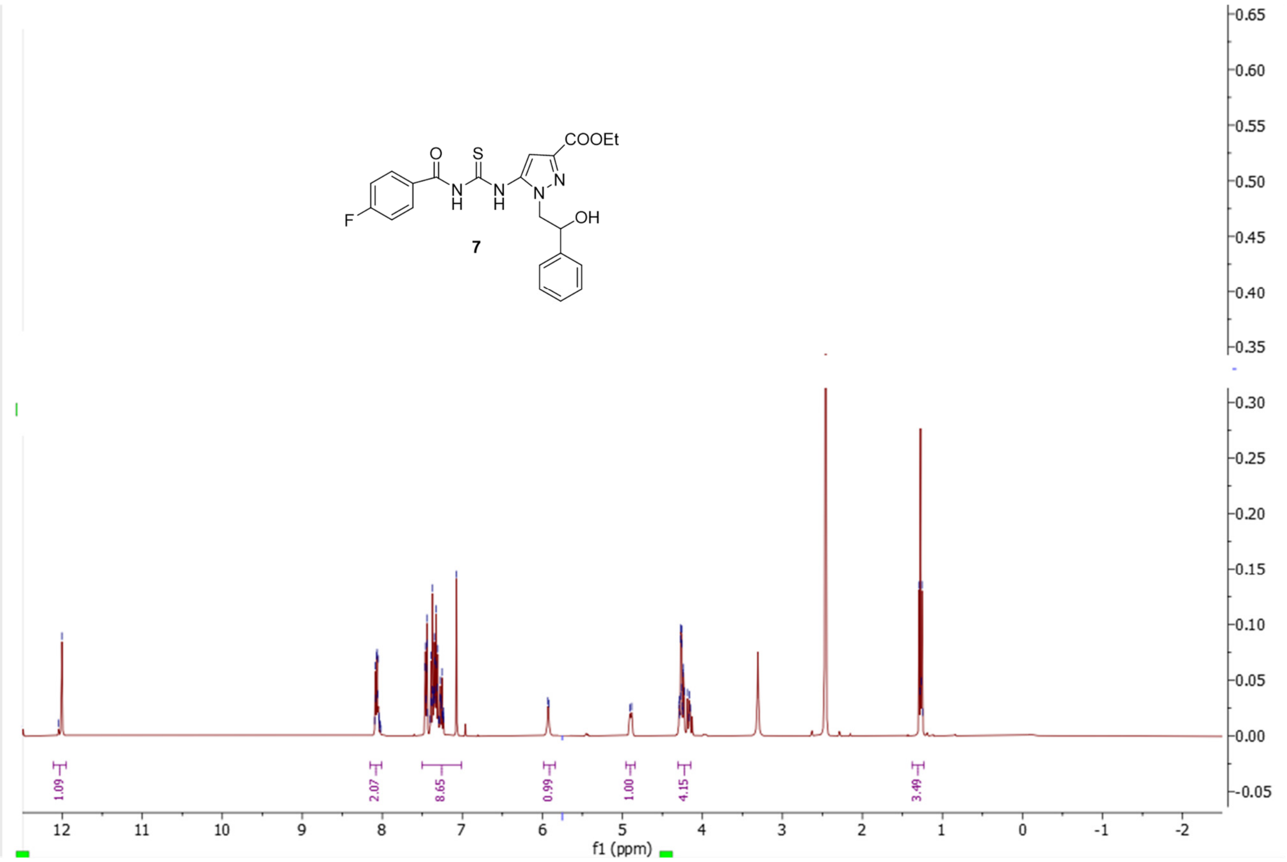

Figure S14. ^{13}C NMR of **7**.

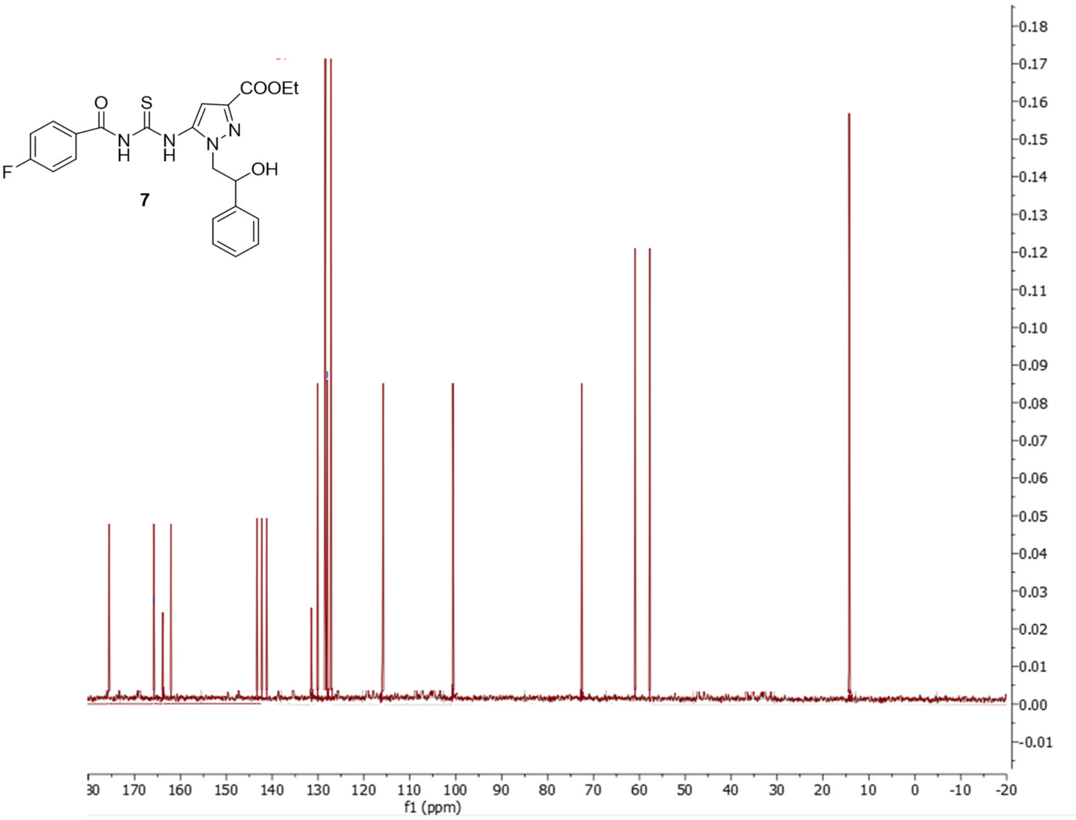

Figure S15. IR of **7.**

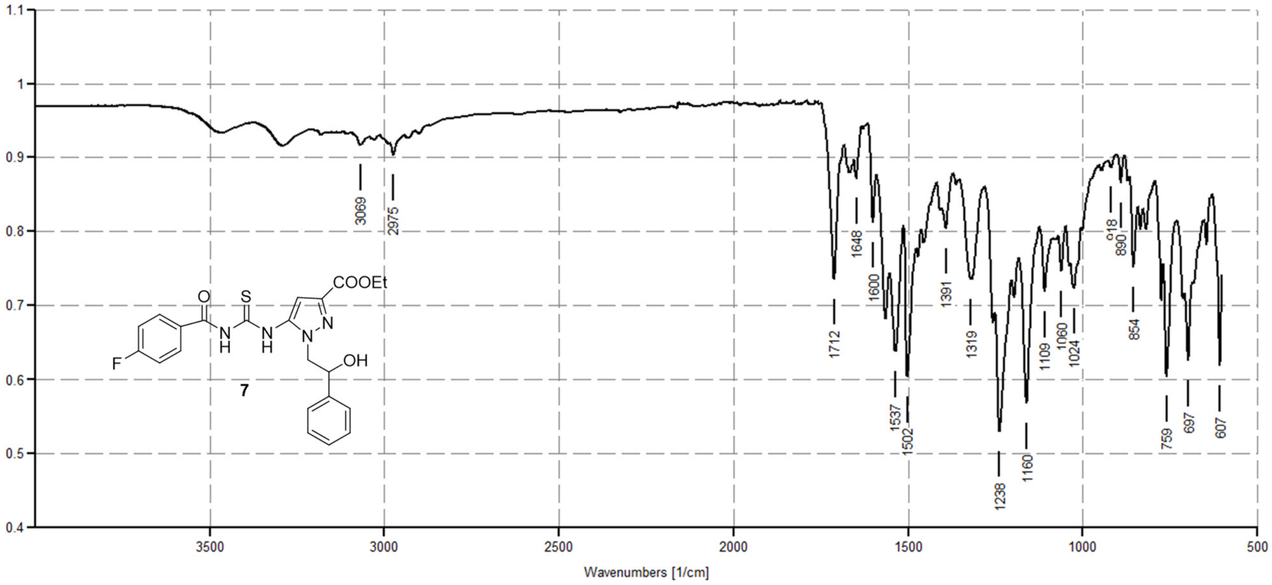

Supplement: Supplementary file 1 [file pharmaceutics-14-01770-s001.zip › pharmaceutics-1859770-supplementary.pdf]
